# Evaluation and Association of Meconium-Stained Amniotic Fluid With Fetal Distress

**DOI:** 10.7759/cureus.83295

**Published:** 2025-05-01

**Authors:** Nilesh S Karpe, Manjusha B Tagad, Rahul R Holkar, Vibha S More

**Affiliations:** 1 Department of Obstetrics and Gynaecology, Dr. Balasaheb Vikhe Patil Rural Medical College, Pravara Institute of Medical Sciences, Loni, IND; 2 Department of Obstetrics and Gynaecology, Seth Gordhandas Sunderdas Medical College and King Edward Memorial Hospital, Mumbai, IND; 3 Department of Pediatrics, Dr. Balasaheb Vikhe Patil Rural Medical College, Pravara Institute of Medical Sciences, Loni, IND; 4 Department of Obstetrics and Gynaecology, Lokmanya Tilak Municipal Medical College, Mumbai, IND

**Keywords:** antenatal, fetal distress, intra-natal, meconium-stained amniotic fluid, obstetric risk factors, stages of labour

## Abstract

Background

Meconium-stained amniotic fluid (MSAF) often leads to complicated deliveries ranging from instrumental delivery, cesarean delivery, and neonatal complications like fetal distress, neonatal intensive care unit (NICU) admission, and neonatal death. Therefore, this study aimed to evaluate the prevalence of MSAF in fetal distress, to determine the clinical profile of newborns in terms of risk factors, and to study the association between antenatal or intra-natal risk factors with MSAF, between umbilical cord pH and MSAF, between stage of labor and MSAF, and between fetal distress and MSAF.

Methods

A total of 200 cases were enrolled after the diagnosis of fetal distress in the intrapartum period in this observational, non-interventional, and cross-sectional study. The inclusion criteria involved pregnant women who had intrapartum fetal distress diagnosed by the abnormal fetal heart rate findings on cardiotocography (CTG) and the interpretation of CTG, adapted from the National Institute for Health and Care Excellence (NICE) Clinical Guideline 190, was used to classify CTG as abnormal irrespective of underlying causes, mode of delivery by cesarean section, and who were willing to participate. The retrospective and prospective data were collected from the labor room records, and statistical analysis was conducted.

Results

The study aimed to evaluate the prevalence of MSAF in fetal distress, for which 52/200 (26%) women were reported with moderate MSAF as an obstetric risk factor. A total of 66/200 (33%) women showed a significant association of antenatal or intra-natal risk factors with MSAF. However, an association between umbilical cord pH and MSAF, and between stage of labor and MSAF, was found to be insignificant. Fetal distress was reported to be significantly associated with MSAF, with 41/200 (20.5%) women reporting variable deceleration and 20/200 (10%) reporting late deceleration.

Conclusion

Moderate MSAF is accounted for as an obstetric risk factor. The association of MSAF with antenatal or intra-natal risk factors was also reported to be significant for obstetric risk factors. But, an association between umbilical cord pH and MSAF, and between stage of labor and MSAF, was found to be insignificant. However, the association between MSAF and fetal distress was significant, indicating the increasing number of cesarean sections being performed for fetal distress. The study reports the association, whereas the future scope might inculcate the strength of the association using odds ratio or prevalence ratio.

## Introduction

The fluid that surrounds the fetus in the uterus is called amniotic fluid, providing a low-resistance and safe environment for the fetus. Meconium is a dark green fluid that the newborn baby passes naturally and comprises epithelial cells, mucus, and bile. Meconium enters the amniotic fluid when the fetus is under stress. It leads to higher perinatal morbidity and death and is an indication of fetal impairment [1]. A higher incidence of instrumental delivery, cesarean delivery, low birth weight, fetal distress, neonatal intensive care unit (NICU) admission rate, and neonatal death is linked to meconium-stained amniotic fluid (MSAF) [2].

Typically, 13% to 16% of childbirths are complicated by MSAF [3]. About 2% to 10% of all cases of MSAF involve meconium aspiration syndrome (MAS), which happens when the newborn aspirates the meconium [4]. In approximately 12% of newborns with MAS, neonatal mortality occurs [1]. MSAF could be a sign of the gastrointestinal tract's typical maturation. It could also exist when there is fetal distress brought on by an acute or ongoing hypoxic episode [4,5]. There has been much discussion over the biology of aberrant amniotic fluid staining, and its clinical implications are still unclear [6]. The green coloration associated with meconium and its consistency leads to a meconium plug and the ability to trigger inflammation in MSAF, a typical anomaly of amniotic fluid staining [7].

The prenatal transfer of meconium from the fetal digestive tract to the amniotic fluid is the subject of several theories. Meconium passing is thought to be a normal physiological phenomenon of the fetus's and its gastroenteric nerve system's growth [5]. However, the contrasting theory states that meconium ejection is a reaction to fetal discomfort. Hypoxia triggers the vagal reflex and vasoconstriction, which in turn causes hyperperistalsis [7]. With conflicting findings, studies have tried to confirm the link between meconium transit in pregnancy and fetal discomfort. According to several studies, MSAF and fetal hypoxia/acidosis are interlinked [8,9]; however, others have not found a connection, indicating that meconium passing during pregnancy is not likely to be linked to worse perinatal outcomes [10,11]. Hence, this study aimed to evaluate the prevalence of MSAF in fetal distress, to determine the clinical profile of newborns in terms of risk factors, and to study the association between antenatal or intra-natal risk factors with MSAF, between umbilical cord pH and MSAF, between stage of labor and MSAF, and between fetal distress and MSAF.

## Materials and methods

This observational, non-interventional, cross-sectional study is part of a large research project performed at the department of obstetrics and gynecology of a tertiary care hospital from January 2018 to December 2019 after obtaining approval from the Institutional Ethical Committee with reference number EC/24/2018. The patients were enrolled after diagnosis of fetal distress in the intrapartum period for the data collection. The diagnosis of fetal distress was made by the abnormal fetal heart rate monitoring findings on cardiotocography (CTG), and the interpretation of CTG, adapted from the National Institute for Health and Care Excellence (NICE) Clinical Guideline 190, was used to classify CTG as abnormal. The inclusion criteria involved singleton pregnancy, gestational age at term (≥30 weeks), cephalic presentation, mode of delivery by cesarean section, as well as term delivery, abnormal fetal heart rate monitoring findings suggestive of fetal distress, and women who were willing to participate. The exclusion criteria included multiple gestations, breech presentation, known fetal anomalies, gestational age less than 30 weeks, intrauterine fetal demise, antepartum hemorrhage, and women who did not want to participate in the present study. The patients were counseled, and those willing to participate in the study were recruited after obtaining their informed consent after the patient delivered the neonate and was shifted to the ward. Based on the eligibility criteria and sample size calculation, a total of 200 cases were enrolled for the present study. The sample size was calculated assuming a desired effect size of 0.3, a significance level (α error) of 0.05, power (1-β error) of 0.92, and degree of freedom (df) of 5. It derived the sample size of 196 participants with actual power of 0.9212 and critical χ² of 11.07, which was rounded off to 200 participants.

The retrospective and prospective data were collected from the labor room records in the data collection sheet and recorded in an Excel sheet (Microsoft Corporation, Redmond, WA). The data were analyzed using SPSS version 17 (SPSS Inc., Chicago, IL). All the parameters were assessed using descriptive statistics, and qualitative data were represented as frequency and percentage. The association was determined using a chi-square test, and a p-value equal to or less than 0.05 was considered significant.

## Results

An observational cross-sectional study was conducted to evaluate the prevalence of MSAF in fetal distress, to determine the clinical profile of newborns in terms of risk factors, and to study the association between antenatal or intra-natal risk factors with MSAF, between umbilical cord pH and MSAF, between stage of labor and MSAF, and between fetal distress and MSAF. The sociodemographic characteristics of the enrolled women are demonstrated in Table 1.

**Table 1:** Sociodemographic characteristics of the enrolled women.

Sociodemographic characteristics (N = 200)		Frequency (n)	Percentage (%)
Age	<20 years	15	7.5%
	21-25 years	84	42%
	26-30 years	74	37%
	31-35 years	22	11%
	36-40 years	5	2.5%
Gravida	Primigravida	107	53.5%
	Multigravida	93	46.5%
Gestational age	30-32 weeks	1	0.5%
	32.1-36 weeks	16	8%
	36.1-38 weeks	55	27.5%
	38.1-40 weeks	65	32.5%

Moreover, the distribution of women amongst various obstetric risk factors is demonstrated in Table 2.

**Table 2:** Distribution of women amongst various obstetric risk factors. MSAF: meconium-stained amniotic fluid; LSCS: lower-segment cesarean section; IUGR: intrauterine growth restriction; PROM: premature rupture of membrane.

Obstetrics factor (n = 200)	Frequency	Percentage (%)
Moderate MSAF	52	26%
Post term	34	17%
Post LSCS	30	15%
Oligohydramnios	24	12%
IUGR	20	10%
PROM	16	8%
Cord around the neck	14	7%
Thick meconium	16	34%
Cord prolapse	1	0.5%
Preterm labor	1	0.5%
Abruptio placenta	1	0.5%
Placenta previa	1	0.5%

The distribution of the women based on MSAF stated that 68 (34%) were affected by meconium and 132 (66%) were not affected by meconium. Moreover, the association between antenatal or intra-natal risk factors with MSAF is demonstrated in Table 3.

**Table 3:** Association between antenatal or intra-natal risk factor and meconium-stained amniotic fluid. * Significant p-value.

Antenatal and intra-natal risk factors	Meconium-stained amniotic fluid		Chi-square test	p-value
	Yes (%)	No (%)		
Obstetrics	66 (33%)	98 (49%)	14.2707	0.0007*
Maternal	0 (0%)	25 (12.5%)		
Both	2 (1%)	9 (4.5%)		
Total	68 (34%)	132 (66%)		

The association between umbilical cord pH and MSAF is demonstrated in Table 4.

**Table 4:** Association between umbilical cord pH and meconium-stained amniotic fluid.

Umbilical cord analysis	Meconium-stained amniotic fluid		Chi-square test	p-value
	Yes (%)	No (%)		
<7	0 (0.0%)	6 (3%)	1.2563	0.2623
≥7	68 (34%)	126 (63%)		
Total	68 (34%)	132 (66%)		

The association between the stage of labor and MSAF is demonstrated in Table 5.

**Table 5:** Association between stage of labor and meconium-stained amniotic fluid.

Stage of labor	Meconium-stained amniotic fluid		Chi-square test	p-value
	Yes (%)	No (%)		
First stage	64 (32%)	127 (63.5%)	0.458	0.499
Second stage	4 (2%)	5 (2.5%)		
Total	68 (34%)	132 (66%)		

Moreover, the association between fetal distress with the sub-groups, mentioning the several criteria to classify fetal distress, including bradycardia, late deceleration, loss of variability, persistent fetal tachycardia, and variable decelerations on CTG and MSAF, is demonstrated in Table 6.

**Table 6:** Association between fetal distress and meconium-stained amniotic fluid. * Significant p-value.

Fetal distress	Meconium-stained amniotic fluid		Chi-square test	p-value
	Yes (%)	No (%)		
Bradycardia	0 (0%)	28 (14%)	14.6219	0.005*
Late deceleration	20 (10%)	30 (15%)		
Loss of variability	3 (1.5%)	5 (2.5%)		
Persistent fetal tachycardia	4 (2%)	9 (4.5%)		
Variable deceleration	41 (20.5%)	60 (30%)		
Total	68 (34%)	132 (66%)		

## Discussion

The present study evaluated the prevalence of MSAF in fetal distress and determined the association of the clinical profile of newborns in women with fetal distress, having an age range of 21 to 25 years. Similarly, a previous study by Gangwar et al. [12] observed that fetal distress was common at 24.5 years of age, and Ajah et al. [13] reported that the average age of women with fetal distress was between 25 and 29 years. Hence, the findings from the above-mentioned studies correspond well with the results of the present study. Moreover, the majority of the patients in the present study were primigravida, which was found to be congruous with the findings of the studies by Gangwar et al. [12] and Ajah et al. [13].

Additionally, fetal distress was more commonly observed in 38.1-40 weeks of gestation, which correlated well with the findings provided by Ajah et al. [13], in which all of the pregnant women were at term with the mean gestational age of 39 ± 2 weeks whereas, in a study given by Ugwa et al. [14] the mean gestational age was 37.6 ± 2.4 weeks, which corresponded with the findings of the current results.

In the present study, moderate MSAF affected 26% of patients out of 200 patients, and in a study conducted by Tasew et al. [15], MSAF affected 23.9% of patients. However, in a study by Aslam et al. [16], there were 19.5% of patients with MSAF out of 123 patients. In the present study, premature rupture of membrane (PROM) affected 8% of 200 patients, whereas Aslam et al. [16] reported that the PROM affected 33.3% of cases out of 123 patients, and the confidence interval was 3.75 to 22.81. However, Gebreheat et al. [17] reported that the PROM affected 4.51 % of 421 patients. A total of 24% of oligohydramnios patients were reported in the present study. However, Tasew et al. [15] reported 4.2% out of 264 patients, and Aslam et al. [16] reported 7.3% cases out of 123 patients under the present findings. One patient (0.5%) reported antepartum hemorrhage out of 200 cases of obstetric high-risk factors in this study. Aslam et al. [16] reported 3.3% out of 123 patients, and Tasew et al. [15] reported 9.1% out of 264 patients. One patient (0.5%) reported cord prolapse out of 200 cases of obstetric high-risk factors in this study. However, Aslam et al. [16] reported 8.1% out of 123 patients, and Tasew et al. [15] reported 8% out of 264 patients. In Gebreheat et al.'s [17] study, cord prolapse affected 4.26% of 421 patients.

Tasew et al. [15] found that the risk of birth asphyxia was 7.9 times higher for newborns with meconium stains than for those without birth asphyxia and that it was 2.2 times more likely to occur in preterm infants than in term ones. Likewise, there was a noteworthy correlation between the neonate's weight and delivery asphyxia. The risk of asphyxia was 6.9 times higher for low birth weight than for normal weight (≥2500 g). This could be because maternal complications such as diabetes mellitus or hypertension, which occur before or during pregnancy, cause low birth weight. Compared to term newborns, preterm babies had a 2.2-fold higher risk of asphyxia. The present study reported no cases of MSAF with cord pH less than 7, indicating that severe fetal acidosis was not observed among these cases; however, 34% of cases were observed with a cord pH more than and equal to 7, hence, suggesting that a significant proportion of fetuses exposed to meconium-stained fluid maintained a normal or near-normal acid-base status at birth. This finding implies that the presence of MSAF alone may not reliably predict severe fetal hypoxia or acidosis. In Kumar et al.'s [18] study, umbilical cord blood pH was the best indicator of fetal hypoxemia during labor, and a pH less than 7 had a higher percentage of NICU transfer. Also, in the present study, it was found that the presence of thick meconium in amniotic fluid is associated with poor fetal outcome and acidosis in comparison to thin meconium-stained liquor.

In the present study, it was observed that there were more cases of MSAF with fetal distress in the second stage of labor (2%) than in the first stage of labor (32%). Lee et al. [19] reported that MSAF has more commonly occurred in the first stage of labor than in the second stage of labor in nulligravida. According to this study, the longer the duration of labor, the higher the chances of MSAF. The second stage of labor is characterized by an increasing number and intensity of uterine contractions compared to the first stage of labor, as well as an increase in maternal bearing down efforts, which leads to maternal fatigue and high fetal lactic acid levels. Maanongun et al. [20] found that out of 140 cases in both the first and second stages of labor, the normal fetal outcome was present in 133 and 123 in the first and second stages of labor, respectively.

Limitations of the study

The sample size could be estimated with broader geographical consideration for the population. The study represents a lack of randomization, providing sampling bias affecting the generalizability of the population. The study incorporated retrospective and prospective data, which could limit the data availability. The study duration can be expanded to determine the long-term association of fetal distress.

## Conclusions

The study concluded that amongst the obstetric risk factors, moderate MSAF accounted for the maximum frequency of women. However, the association of MSAF with antenatal or intra-natal risk factors was significant concerning obstetric risk factors, but that with umbilical cord pH and stage of labor was insignificant. Also, the association between MSAF and fetal distress was significant, specifying that the presence of meconium in the amniotic fluid is a meaningful clinical sign of potential fetal compromise, even if not always accompanied by changes in cord blood pH. The study reports the association, whereas the future scope might inculcate the strength of the association using odds ratio or prevalence ratio.
